# Development of Novel Cornstarch Hydrogel-Based Food Coolant and its Characterization

**DOI:** 10.3390/polym16050569

**Published:** 2024-02-20

**Authors:** Yalu Zheng, Yan Ma, Ruchika Hansanie Ukwatta, Feng Xue, Chen Li

**Affiliations:** 1 College of Food Science and Light Industry, Nanjing Tech University, Nanjing 211816, China; 2 School of Pharmacy, Nanjing University of Chinese Medicine, Nanjing 210023, China

**Keywords:** cornstarch, food coolant, freeze–thaw cycle

## Abstract

The food, pharmaceutical, and supply transport storage chain is seeking coolants that come with plastic-free packaging, are nontoxic, environmentally friendly, robust, reusable, and reduce water waste. To meet this demand, a new food coolant based on cornstarch hydrogel was developed and tested using the regeneration method. This study investigated the reusability, water retention, rehydration, and surface cleanliness of the hydrogel, along with its application in freshness retention for fruits. The results of the gel strength and differential scanning calorimetry (DSC) analysis showed that the ideal concentration of cornstarch hydrogel was 8%. Freezing and thawing experiments demonstrated that the hydrogel had the potential to be used as a cooling medium for refrigerated fresh foods. Moreover, the gel strength, scanning electron microscopy images (SEM), DSC, and thermogravimetric analysis (TG) revealed that the freeze–thaw reuse only slightly affected its freezable water content and that its gel strength gradually increased during reuse. Water retention and rehydration tests showed that the hydrogels could be better preserved at −20 °C compared to 4 °C, and the water lost during reuse could be replenished through rehydration. The flexibility in terms of shape and size also allows the hydrogel ice to be used as a customized coolant for various food shapes, as demonstrated by preservation experiments. Additionally, washing the hydrogel after each use can result in a significant reduction in *Escherichia coli*, *Salmonella*, and *Staphylococcus aureus* concentrations by 3.03, 3.47, and 2.77 log CFU/hydrogel, respectively. Overall, the new cornstarch hydrogel coolant is a promising alternative to conventional ice, with the potential to serve as a food coolant.

## 1. Introduction

As people’s expectations for food quality have increased, the standards for food freezing and storage have also become more stringent. Food coolants are used to lower the temperature of food products to refrigeration levels. Industry commonly uses cold air cooling, cold water cooling, contact ice cooling, and vacuum cooling for food preservation [1]. In certain condition-constrained transportation storage scenarios, ice is the most readily available and commonly used cooling medium by customers and retailers. Despite its benefits, such as fast cooling, affordability, harmlessness, portability, and effectiveness in food preservation, there are significant drawbacks to using ice. The extensive use of ice cubes leads to significant water waste, as they are not reusable. The meltwater from ice cubes can easily contaminate fresh food, leading to cross-contamination. Moreover, once the ice cubes melt, they no longer support the food, which increases the risk of direct contamination from the outside environment.

An ideal food coolant should possess certain key characteristics, such as high cooling efficiency, reduced meltwater, reduced cross-contamination, sustainability, reusability, biodegradability, biocompatibility, and be free of plastic. Zou et al. [2] demonstrated that protein-based hydrogels can be used as a novel coolant to achieve these goals. The term “hydrogel” first appeared in the literature in 1894 [3]. Hydrogels are gels with a three-dimensional (3D) network structure formed by hydrophilic or water-soluble polymers through physical or chemical cross-linking, wherein water is the dispersion medium and accounts for at least 70% of the weight of the gel, thereby possessing strong water retention capabilities. To date, no other ideal coolant has been discovered.

We propose the novel idea of using a polysaccharide hydrogel as a replacement for conventional ice cubes in food cooling. To the best of our knowledge, there has been no previous use of polysaccharide hydrogels as food coolants. Polysaccharides and their derivatives possess desirable properties such as biodegradability, eco-friendliness, abundance in source, and low cost, which make them ideal matrix materials for hydrogels [4]. Currently, hydrogels based on polysaccharides like chitosan, alginate, cellulose derivatives, pectin, and starch have been extensively researched and utilized [5,6,7,8]. We aim to explore the potential of polysaccharide-based hydrogels as food coolants.

Cornstarch was used as the raw material for preparing hydrogels due to its abundance and low cost. Cornstarch contains numerous hydroxyl groups that facilitate cross-linking reactions, making it an ideal material for constructing natural biomacromolecule-based hydrogels [9]. During cornstarch gelatinization, its molecular chains interact, entangle, and rearrange via hydrogen bonding, resulting in a starch hydrogel network through physical cross-linking and the orderly formation of microcrystals in some regions [10,11]. Thus, the formation of cornstarch hydrogel is caused by the rearrangement of straight chain starch during heating and cooling. Based on the structure, an ideal new food coolant can be developed. Furthermore, the incremental cost-effectiveness of this new coolant is significant not only because of the fact that cornstarch is rich in raw materials, inexpensive, and easy to prepare but also the coolant was developed to be suitable for repeated use, thereby reducing the maintenance and replacement costs. Last, but not least, the new coolant is of food grade and environmentally friendly, which complies with environmental regulations, therefore serving as the basis for developing new cooling products.

In this study, the cornstarch hydrogels were prepared, and the optimal concentration served as the ideal coolant was evaluated. Subsequently, the reusability of the hydrogels was examined through a series of freeze–thaw cycle (FTC) experiments. Moreover, the application of hydrogel ice on fresh fruit and the surface cleaning ability were evaluated. The ultimate goal of this research is to develop a new hydrogel ice to replace traditional ice and address its advantages associated with food preservation.

## 2. Materials and Methods

### 2.1. Materials and Reagents

Cornstarch ((C_6_H_10_O_5_)_n_, purity—98%, 74.3 wt.% of amylopectin and 25.7 wt.% of amylose, M_w_ 1.50 × 10^7^) was purchased from Shanghai Yuanye Biotechnology Co., Ltd. (Shanghai, China). Sodium chloride was purchased from Beijing InnoChem Science & Technology Co., Ltd. (Beijing, China). Luria Bertani (LB) Broth and Plate Count Agar (PCA) were purchased from Qingdao Hi-Tech Industrial Park Hope Bio-Technology Co., Ltd. (Qingdao, China).

### 2.2. Preparation of Hydrogels

Cornstarch hydrogels (4, 6, 8, and 10%, *w/v*) were prepared according to the methods of Srichuwong et al. [12] and Zou et al. [2], with minor modifications. Firstly, different concentrations of cornstarch solutions (6–10%, *m/v*) were prepared and stirred continuously with a magnetic stirrer (HJ-4, Changzhou, China), and the solutions were heated to 95 °C for 5 min, then cooled down to 25 °C at 5 °C/min. To prevent water evaporation, the beaker was covered with tin foil during the heating and cooling treatment. Finally, a 7 g starch solution was poured into the mold (2 cm × 2 cm × 2 cm). The mold was placed in a 4 °C refrigerator for 24 h to induce the gel formation.

### 2.3. FTC of Hydrogels

The hydrogels prepared in Section 2.2 were subjected to FTC, i.e., stored at −20 °C for 18 h and then thawed at room temperature (25 °C) for 6 h, which was named as one cycle of the freeze–thaw treatment. A total of five freeze–thaw cycles (FTC0–5) were performed. At the same time, deionized water was used as a control.

### 2.4. Texture Determination

The hardness of the hydrogel after FTC0, FTC1, FTC3, and FTC5 was determined by a texture analyzer (TMS-Pro, Food Technology Corporation, Sterling, VA, USA) with a 50 mm cylindrical probe. The measurement was performed with an initial force of 0.5 N at a speed of 60 mm/min and 30% of deformation. The average value was taken after 3 parallel measurements for each group of samples.

### 2.5. Melting Latent Heat

The latent heat of melting was analyzed using differential scanning calorimetry (DSC) (Q-20, TA Instruments, New Castle, DE, USA) according to the method of Zou et al. [2]. The endothermic release curves of the hydrogels from −30 °C to 10 °C were measured at a heating rate of 1 °C/min under nitrogen with a flow rate of 50 mL/min. The melting latent heat of hydrogel at 0 °C was calculated by integrating the heat flow (W/g) versus time (s) on the curve.

### 2.6. The Measurement of the Cooling Rate

The colling rate was measured according to Seetapan et al. [13], with minor modifications. The probe of the thermocouple was placed in the center of the starch solution to record the changes in temperature during the freezing treatment. The freezing rate was calculated according to Equation (1) [14].

(1) $$Freezing\;rate({}^{\circ}C/min)=\frac{T_{2}-T_{1}}{t_{2}-t_{1}}$$

where T_1_ and T_2_ represent the initial freezing temperature and final freezing temperature, respectively, and t_2_ and t_1_ represent the freezing start time and ending time, respectively.

### 2.7. Melting Curves

The changes in temperature during the melting were also recorded by using the thermometer. The temperature was recorded to 10 °C, as it was widely used to simulate the temperature for food preservation by normal ice.

### 2.8. Scanning Electron Microscope (SEM) Observation

The microstructure of the samples was measured by Seetapan et al. with minor modifications [13]. The hydrogel was freeze-dried, and then, the central section was plated with gold by ion sputtering. The microstructure was observed under a scanning electron microscope (GeminiSEM 500, ZEISS, Jena, Germany) at an accelerating voltage of 5 kV.

### 2.9. Thermogravimetric Analysis (TGA)

The weight loss of the samples was obtained from 30 °C to 200 °C at 10 °C/min using a thermogravimetric analyzer (TG209F1, NETZSCH Corporation, Selb, Germany) under nitrogen. The derivative thermogravimetric analysis (DTG) curves were obtained from the reaction rates and used to help identify the free and bound water volumes.

### 2.10. Water Retention Measurement

The water retention capacity of the hydrogel was measured according to Zou et al. [2]. In brief, the hydrogel was placed at 4 °C and −20 °C, respectively. The weight changes were measured to evaluate the water-holding capacity of hydrogel ice.

### 2.11. Rehydration Capacity

The hydrogel was immersed in 50 mL of deionized water for 20 min after FTC and then frozen again. The absorption of water and properties were measured to evaluate the reusability of the hydrogel ice.

### 2.12. Cooling Curve of Fresh Grapes and Blueberries

The grapes (6 g) or blueberries (1 g) were placed on large hydrogel ice (8 g, 2 cm × 2 cm × 2 cm) or small hydrogel ice (1 g, 1 cm × 1 cm × 1 cm) and left for a period of time, and the temperature change was measured with a thermometer. The measurements were performed in an incubator.

### 2.13. Surface Cleaning

To characterize the cleaning ability, the (2 cm × 2 cm × 2 cm) hydrogel was first irradiated under a UV lamp for 30 min to remove surface bacteria. Then, 100 µL of *Escherichia coli* (1.643 × 10^7^), *Salmonella* (2.12 × 10^7^), and *Staphylococcus aureus* (1.53 × 10^7^) were inoculated on the surface of the hydrogel for about 1 min, and the contaminated hydrogel was washed twice with 10 mL of deionized water. Finally, the hydrogel was rinsed with 10 mL of saline (0.85%), and the remaining bacteria on its surface were counted.

### 2.14. Statistical Methods

Each experiment was performed at least three times, and the results were described as the mean ± square deviation (SD). The data were analyzed using SPSS 26.0 software, and different superscripts of values represented significant differences (*p* < 0.05).

## 3. Results

### 3.1. Determination of the Concentration

**Figure 1:**
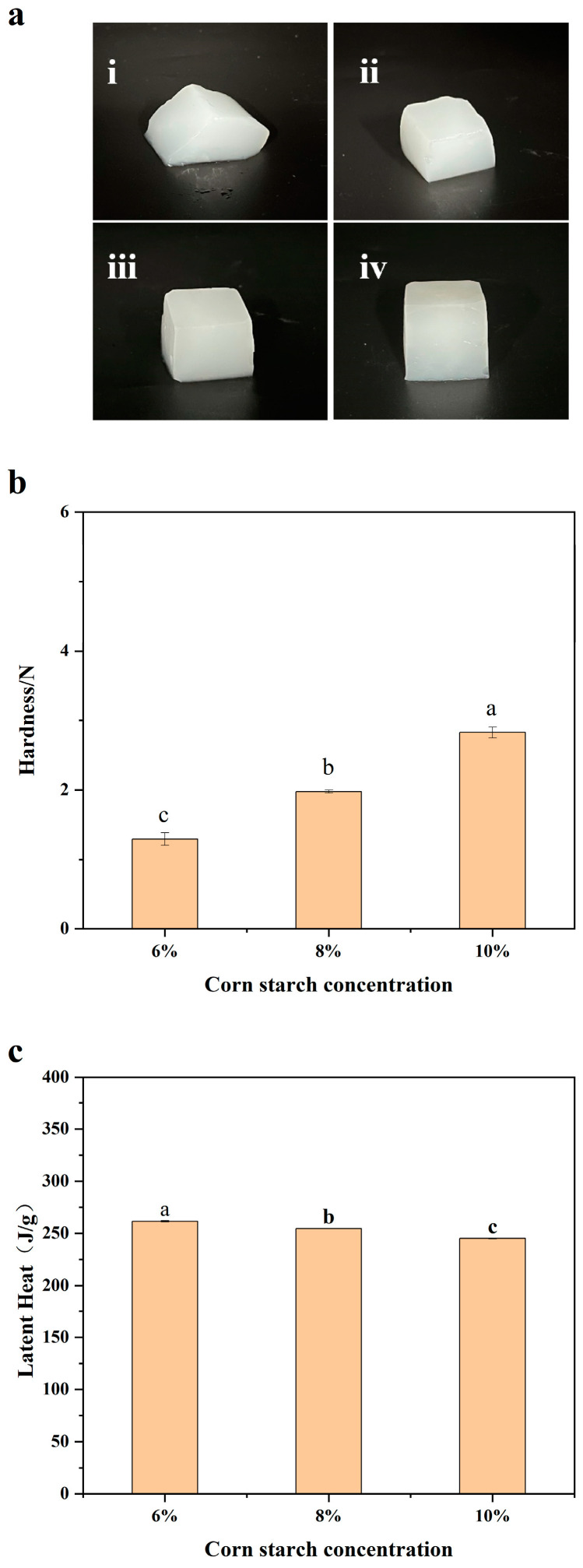
(**a**) The appearance of hydrogels prepared by different concentrations of cornstarch: 4% (**i**), 6% (**ii**), 8% (**iii**), and 10% (**iv**). (**b**) Hardness of hydrogels with different cornstarch concentrations. (**c**) Latent heat diagram of the melting of cornstarch hydrogels. Results having different letters (a–c) are significantly different (*p* < 0.05).

The concentration of starch in hydrogel plays a crucial role in determining its gel properties. The hardness of the hydrogel increases with the starch concentration due to the higher amount of amylose and longer amylopectin chains [15]. Figure 1a shows that a 4% of cornstarch concentration is inadequate for practical use, and cornstarch concentrations above 6% are sufficient to form stable and noncollapsing hydrogels. A previous study showed that the critical concentration for cornstarch to form hydrogel is around 6% [16].

Figure 1b indicates that the hardness of hydrogel increases with an increase in the starch concentration. A gel that can withstand a pressure of 2 N is considered a high-strength gel suitable for carrying ordinary food. According to Figure 1b, the concentration of starch should be higher than 8% to achieve the required mechanical strength.

The latent heat of melting of cornstarch hydrogels with different concentrations was determined using differential scanning calorimetry (DSC). As shown in Figure 1c, the latent heat of melting of cornstarch hydrogel increases with the increasing water content, indicating that a higher water content ensures a higher heat absorption capacity. Although a cooling effect of hydrogel ice comparable to that of ordinary ice can be achieved by increasing the amount, its practical application in reducing the amount of water and cross-contamination of food caused by melting water is also of great importance. The average latent heat of the melting of 8% cornstarch hydrogel was 254.6 J/g, which was 76.1% of the latent heat of conventional ice (334.5 J/g). Therefore, a concentration of 8% is considered as the most suitable concentration for practical use. The following experiments are based on the concentration of 8%.

### 3.2. Temperature Profiles of Freezing and Melting Times

**Figure 2:**
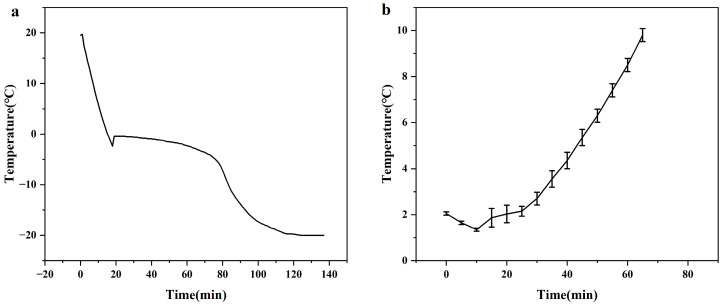
(**a**) Freezing curve of hydrogel ice. (**b**) Melting curve of hydrogel ice.

The cornstarch hydrogel samples were cryogenically frozen to start the freeze–thaw cycle. Figure 2a illustrates the time–temperature freezing curve of the hydrogel. During the supercooling phase, the temperature drops from 20 °C to below the initial freezing point and then rises back up to the initial freezing point. The system enters the phase change when the temperature rises from the point where ice crystallization starts [17]. At the end of the phase change stage, the temperature slowly drops to the storage temperature. The hydrogel ice was frozen for 83 ± 5 min at a freezing rate of −0.25 ± 0.1 °C/min. A faster freezing rate leads to the formation of smaller ice crystals during the sample freezing, which causes less damage to the internal structure and results in a more uniform size of the internal structure [18]. Figure 2b shows the melting curve of hydrogel ice, which can maintain a temperature of 0–10 °C for 60 min for a 2 cm × 2 cm × 2 cm cube, making it suitable as an alternative to traditional ice for preserving fresh food.

### 3.3. Properties of Hydrogels along FTCs

**Figure 3:**
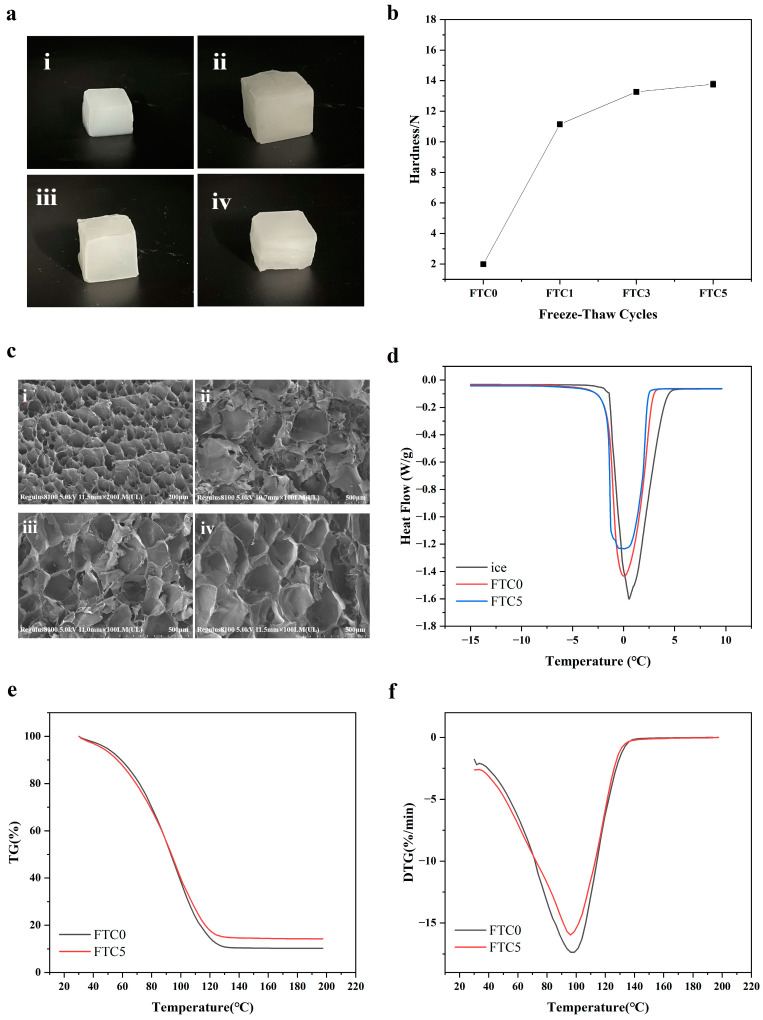
(**a**) Photo of the hydrogels after FTC0 (**i**), FTC1 (**ii**), FTC3 (**iii**), and FTC5 (**iv**). (**b**)Hardness of the hydrogels after different numbers of freeze–thaw cycles. (**c**) SEM images of the hydrogels after FTC0 (**i**), FTC1 (**ii**), FTC3 (**iii**), and FTC5 (**iv**). (**d**) DSC curves of the ice and hydrogels after FTC0 and FTC5. Thermogravimetric curves (**e**) and derivative thermogravimetric curves (**f**) of the hydrogels after FTC0 and FTC5.

The reusability of cornstarch hydrogel ice was evaluated by subjecting them to repeated freeze–thaw treatments. Images of the hydrogel ice cubes were taken after different freeze–thaw cycles, as shown in Figure 3a. The hydrogel ice cubes were found to be homogeneous, translucent, and glossy after FTC0. After FTC1, the color was between translucent and opaque, and a few water droplets were observed. After FTC3, the edges turned white due to water loss, and cracks appeared after FTC5.

The mechanical hardness of the hydrogel ice is a crucial parameter for reusing. As shown in Figure 3b, the mechanical strength of hydrogel ice increases significantly from 2 N to 11.2 N with the increase in freeze–thaw cycles, which is consistent with the reports of Teng et al. and Jing et al. [19,20]. Therefore, the FTC process hardly influences the mechanical strength, which ensures the support of food during subsequent repeated use.

The SEM experiments showed a complete and homogeneous honeycomb structure of FTC0 hydrogel (Figure 3(ci)). Significant differences in the microstructures were observed after FTC1. Water in the hydrogel formed ice chambers when frozen, and starch molecules joined around the ice chambers to form a solid matrix, resulting in the formation of a thick network of starch-condensed fibers. After freeze-drying, the ice chambers disappeared, and large cryogel ice structure cavities were formed, with inhomogeneous pore sizes, as shown in Figure 3(cii). After several freeze–thaw cycles, the cornstarch cryogel formed a sponge-like structure consisting of ice cells and a fibrous network [21], with the starch walls wrapped around the ice crystals being thicker, the voids larger, and the cavities becoming gradually homogeneous, as shown in Figure 3(ciii,civ). This is consistent with the findings of Wang et al. [22]. Moreover, the cavities significantly increase with the increasing number of freeze–thaw cycles, verifying the destructive nature of repeated freeze–thaw treatments on the polymer structure due to the formation of ice crystals through repeated freezing causing pore channels to appear in the starch walls [23]. Therefore, further research is needed to reduce the damage of ice crystals on the structure of cornstarch hydrogel. Approaches such as accelerating the freezing rate [24], adding functional ingredients [25,26,27,28], and introducing chemical cross-linking to strengthen the hydrogel matrix [29] are some feasible solutions to reduce the damage to the polymer structure by ice crystals.

Freezable water of the hydrogel ice was evaluated by DSC analysis. Figure 3d indicates that the phase transition temperature of pure water was higher than that of the cornstarch hydrogel. The hydrogel ice showed a heat absorption peak at around −3 °C, while it began to melt at −6 °C, which is consistent with previous research by Freschi et al. [30]. The lower temperature heat absorption in the hydrogel may be due to the presence of freezable bound water. Furthermore, it was observed that the heat absorbed in the melting ice region of the hydrogels after FTC5 slightly decreased as compared to that after FTC0. The heat absorption area also decreased. These results suggest that freeze–thaw cycles slightly decline the cooling performance of hydrogel ice.

TGA is considered an effective and accurate method for measuring bound water in cornstarch hydrogels [31]. Figure 3e,f shows the TGA and DTG curves of the hydrogels after FTC0 and FTC5. The weight loss is due to the decrease in the total water content, including free and bound water. From Figure 3e, it can be seen that the total water content of the samples decreased with the increase in the number of freeze–thaw cycles. From Figure 3f, it can be seen that the samples lose free water below 100 °C and bound water after exceeding 100 °C. The results suggest that freeze–thaw cycles cause a decrease in the bound water of the hydrogel samples, which therefore decreases the cooling performance of hydrogel ice. The results are consistent with DSC studies.

### 3.4. Water Retention, Rehydration, and Cooling Efficiency

**Figure 4:**
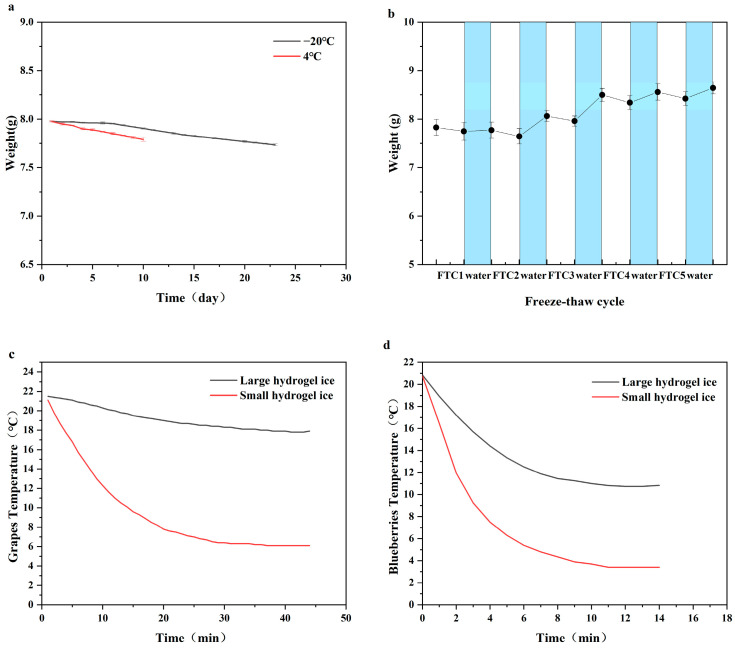
(**a**) Water retention of hydrogels. (**b**) Water loss–rehydration cycle of hydrogels. Large hydrogel ice and small hydrogel ice for preserving grapes (**c**) and blueberries (**d**).

Maintaining the water content for a satisfactory cooling performance is a major challenge for cornstarch hydrogel ice due to the starch regrowth, dehydration, and shrinkage during freezing and long-term storage with the absence of a plastic shell. The water-holding ability of hydrogel ice was tested at −20 °C and 4 °C. The experimental results indicated that hydrogel ice could be stored at 4 °C for up to 10 days before deteriorating and becoming moldy, with 97.7% of the mass remaining after 10 days. In contrast, hydrogel ice stored at −20 °C experienced slow dehydration in the first few days, with only 0.209% mass loss after 6 days in storage, followed by a gradual acceleration in mass loss, with 97% of the mass remaining after 25 days. This finding is consistent with the study of Apostolidis et al., who reported an increase in water exudation along with the storage and a reduction in the water-holding capacity correlated with the hydrogel strength enhancement [15]. Dehydration can lead to impaired cooling performance during freeze–thaw cycles. Additionally, as shown in Figure 4a, hydrogel ice exhibits better water retention at −20 °C than at 4 °C. Therefore, it is advisable to store hydrogel ice at lower temperatures whenever possible to minimize dehydration-induced shrinkage. Rehydration can be performed after each freeze–thaw cycle to compensate for water loss.

Figure 4b indicated that each freeze–thaw cycle results in a mass loss of approximately 1–2%. Rehydration experiments showed that 0.3–6.5% of water can be recovered, leading to the hydrogel weight increasing by 10.38% compared to the initial weight after five cycles. Thus, the water loss of hydrogel ice can be compensated by rehydration, enhancing its cooling capacity. The experiments demonstrated that hydrogel ice can be reusable for cooling food.

In comparison to ice bags, hydrogel ice has the advantage of adjusting the shape and size. Figure 4c,d show that the cooling efficiency varies due to the size of the hydrogel ice. A previous study suggested that a temperature range of 0–10 °C be widely applied for refrigeration [32]. The large pieces of hydrogel ice were unable to cool grapes below 10 °C within 45 min, while small pieces could decrease the grape temperature to 9.6 °C after 15 min, and the temperature further decreased along with the preservation (Figure 4c). For blueberries, as shown in Figure 4d, large hydrogel ice is capable of cooling them to 10.7 °C within 13 min. However, it takes only 3 min for the small hydrogel ice to cool the temperature to 9.2 °C, and the temperature also decreased along with the refrigeration. Therefore, the cooling efficiency of hydrogel ice can be improved by size reduction. The flexibility in shape and size makes hydrogel ice an excellent option as a custom coolant for different food shapes.

### 3.5. Surface Cleaning of Cornstarch Hydrogel Ice

**Figure 5:**
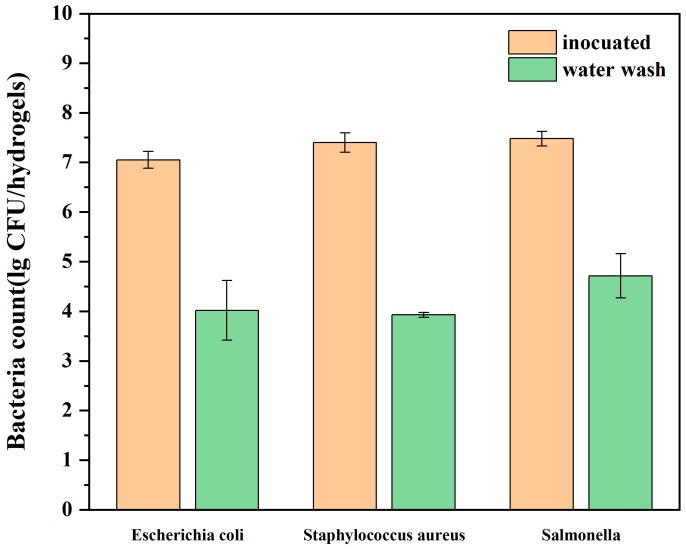
The total number of colonies before and after hydrogel washing.

In order to achieve a reusable food coolant, surface cleaning is crucial for hydrogel ice. Therefore, it is important to investigate whether water washing after each use can reduce the number of microorganisms on the hydrogel surface. As presented in Figure 5, the water washing led to a reduction of 3.03, 3.47, and 2.77 log CFU/hydrogel for *E. coli*, *S. aureus*, and *Salmonella*, respectively. The findings demonstrate that water washing can effectively clean the hydrogel surface. However, it should be noted that the washing process rehydrates the hydrogel; thus, the duration of washing should be carefully controlled to prevent excessive hydration.

## 4. Discussion

The experimental results have demonstrated the potential of cornstarch hydrogel as a new food coolant. This new coolant has several advantages, such as low cost, easy to operate, reusable, sturdy, and environmentally friendly. Additionally, it can prevent the cross-contamination caused by traditional ice melting and reduce water waste. The experiments on grape and blueberry preservation have also proved its cooling effectiveness. Nonetheless, there are still challenges in its application, such as strengthening the hydrogel matrix during preparation to reduce the structural damage caused by freeze–thaw cycles and enhancing its cooling efficiency to match that of conventional ice. Water washing after each use has been found to reduce the number of surface colonies, but care should be taken not to overhydrate the hydrogel during this process. Expanding its functionality is also a priority, and developing pH-responsive or photosensitive antibacterial coolants is a promising direction for future research.

## 5. Conclusions

The ideal concentration of the cornstarch hydrogel was 8%, and the hydrogel has the potential to be used as a cooling medium for refrigerated fresh foods. Furthermore, freeze–thaw cycles could decline the cooling performance of hydrogel ice, while the gel strength gradually increased during reuse. The hydrogels were better preserved at −20 °C, and the water lost during reuse could be replenished by rehydration. The flexibility of hydrogel ice in terms of shape and size also allows it to be used as a customized coolant for a wide range of food shapes, as demonstrated by the preservation experiments. In addition, washing the hydrogel after each use resulted in significant reductions in *E. coli*, *S. aureus*, and *Salmonella* concentrations of 3.03, 3.47, and 2.77 log CFU/hydrogel, respectively. In conclusion, the novel cornstarch hydrogel coolant is a promising alternative to traditional ice. The development of a functional hydrogel ice should be considered for future studies.

## Data Availability

Data are contained within the article.
